# Vanadium Hexacyanoferrate as a High-Capacity and High-Voltage Cathode for Aqueous Rechargeable Zinc Ion Batteries

**DOI:** 10.3390/nano12234268

**Published:** 2022-11-30

**Authors:** Shijing Zhang, Qiang Pang, Yuqing Ai, Wei He, Yao Fu, Mingming Xing, Ying Tian, Xixian Luo

**Affiliations:** 1 School of Science, Dalian Maritime University, Dalian 116026, China; 2 School of Physics and Materials Engineering, Dalian Minzu University, Dalian 116600, China

**Keywords:** high capacity, hybrid electrolyte, vanadium hexacyanoferrate, zinc-ion batteries

## Abstract

Prussian blue analogs (PBAs) are widely used as electrode materials for secondary batteries because of their cheapness, ease of synthesis, and unique structural properties. Nevertheless, the unsatisfactory capacity and cyclic stability of PBAs are seriously preventing their practical applications. Here, vanadium hexacyanoferrate (VHCF) is successfully prepared and used as a cathode for aqueous zinc-ion batteries (AZIBs). When using 3 M Zn(CF_3_SO_3_)_2_ as the electrolyte, a high capacity of ~230 mA h g^−1^ and a high voltage of ~1.2 V can be achieved. The XRD result and XPS analysis indicate that the outstanding Zn^2+^ storage capability is due to the presence of dual electrochemical redox centers in VHCF (Fe^2+^ ⇋ Fe^3+^ and V^5+^ ⇋ V^4+^ ⇋ V^3+^). However, the battery shows a short cycle life (7.1% remaining capacity after 1000 cycles) due to the dissolution of VHCF. To elongate the cycle life of the battery, a high-concentration hybrid electrolyte is used to reduce the activity of water molecules. The improved battery exhibits an impressive capacity of 235.8 mA h g^−1^ and good capacity retention (92.9%) after 1000 cycles.

## 1. Introduction

In recent years, there have been growing calls from various industries to improve battery safety, mainly due to concerns about the fire and even explosion risks of lithium-ion batteries [1,2,3]. Therefore, it is very important to develop alternative high-performance batteries with lower cost and naturally non-explosive properties [4]. Rechargeable aqueous zinc-ion batteries (AZIBs) have recently attracted much attention due to their numerous advantages, especially their excellent safety [5,6,7]. As the anode electrode of AZIBs, zinc metal has many unique merits, such as wide distribution of resources, high stability, high gravimetric capacity, and appropriate electrochemical potential [8,9]. However, the capacity and electrochemical reversibility of the cathode materials are greatly limited due to the poor electrochemical kinetics of Zn^2+^ [10]. Moreover, the practical application of AZIBs is still challenging due to the zinc dendrite growth and loss of active materials in conventional ZnSO_4_ and Zn(CF_3_SO_3_)_2_ aqueous electrolytes [11].

Currently, the properties of manganese-based and vanadium-based cathodes for AZIBs have been widely studied [12,13]. However, the dissolution of Mn2+ from manganese-based cathodes during the charge and discharge usually leads to a fast capacity decay [14]. Although vanadium-based cathodes have a relatively long life cycle, they generally show low operational voltage (<0.9 V), resulting in a low energy density of full batteries [15]. As another promising cathode material for AZIBs, Prussian blue analogs (PBAs) are inexpensive, high-performance, and easy to implement in industrial large-scale synthesis [16,17,18,19]. The general formula of PBAs is A_x_M_1_[M_2_(CN)_6_]_y_·zH_2_O, where the A is the alkaline cations and the M_1_/M_2_ are the transition metal ions. The most typical atom on the M_2_ site is Fe. The FeC_6_ octahedrons and the M_1_N_4_ tetrahedrons are connected through CN ligands, forming a large three-dimensional framework, enabling rapid and highly reversible intercalation/de-intercalation of cations (NH^4+^, Li^+^, Mg^2+^, Zn^2+^, etc.) [20,21]. Moreover, the electrochemical behaviors of PBAs, such as their capacity and operational voltage, can be regulated by controlling their chemical compositions. For instance, Dr. Zhang studied the properties of a zinc//zinc hexacyanoferrate (Zn//ZnHCF) battery in an aqueous electrolyte [22]. The battery delivered a capacity of 65.4 mA h g^−1^ at 1C with a high operational voltage of 1.7 V, and a capacity retention of 76% after 100 cycles at 1C. Later, copper hexacyanoferrate (CuHCF) was studied as a cathode electrode in AZIBs, which showed a lower operational voltage (~1.6 V) and a similar initial capacity (~64 mA h g^−1^) [23]. Recently, a reduced graphene oxide (RGO) modified NiHCF/RGO composite cathode for AZIBs was proposed by Xue and co-workers, giving a good cyclic stability of 1000 cycles (80.3% capacity retention). However, it only showed a limited capacity (47.8 mA h g^−1^) and a low discharge voltage (~1.2 V) [24]. It can be found that, as a cathode electrode for AZIBs, PBAs usually exhibit relatively low specific capacity. This is mainly due to the single electrochemical active site (Fe ions in Fe(CN)6 groups) in these PBAs. A major breakthrough in achieving high-capacity Zn//PBA batteries was achieved by Prof. Zhi and co-workers in 2019. Their proposed cobalt hexacyanoferrate (CoHCF) cathode could realize a two-species redox reaction (Co and Fe), providing a high average voltage of 1.7 V and an impressive capacity of ~170 mA h g^−1^ [25]. Therefore, regulating the composition and electrochemically active species of PBAs may be a feasible method to enhance the performances of Zn//PBA batteries.

Here, vanadium hexacyanoferrate (VHCF) was successfully synthesized by a co-precipitation reaction and applied to AZIBs as a cathode. The VHCF cathode showed a high capacity (~230 mA h g^−1^) after a short activation process. The XPS results indicate that the high capacity of VHCF was due to the existence of dual electrochemical redox centers (Fe^2+^ ⇋ Fe^3+^ and V^5+^ ⇋ V^4+^ ⇋ V^3+^), which can realize a three-electron redox reaction. However, its capacity decayed rapidly as the active material dissolved into the electrolyte (3 M Zn(CF_3_SO_3_)_2_) during cycling. Then, a high-concentration hybrid electrolyte (3 M Zn(CF_3_SO_3_)_2_ + 10 M LiTFSI) was used to improve the cycle stability of the Zn//VHCF battery. This improved battery exhibited a high capacity of 235.8 mA h g^−1^ and a remarkable capacity retention (92.9%) after 1000 cycles.

## 2. Materials and Methods

### 2.1. Synthesis of VHCF

Firstly, 25 mL of 32% hydrochloric acid was diluted to 37.5 mL with deionized (DI) water. Then, 2 g of V_2_O_5_ powders were added into the diluted acid solution with constant stirring to obtain a yellow suspension. After stirring for 1 h, 350 μL of glycerol was added drop by drop under stirring until a clear solution was formed. Then, 4.7 mL of the clear solution was diluted to 25 mL. After this, 0.5926 g K_3_Fe(CN)_6_ was dissolved in 25 mL of DI water. Then, it was slowly added to the diluted solution. After stirring for 24 h, the precipitate was centrifuged and washed six times alternately with DI water and alcohol. Finally, the prepared VHCF sample was dried in the oven at 60 °C for one day.

### 2.2. Materials Characterizations

The structures of the VHCF powder and cathode electrodes were studied by powder X-ray diffraction (XRD) tests using an X-ray diffractometer (D/MAX-Ultima, Shimadzu Corporation, Kyoto, Japan) with Co K_α_ radiation at room temperature. The valence states of the elements in the VHCF cathodes were studied by X-ray photoelectron spectroscopy (XPS) spectra using an X-ray photoelectron spectrometer (ESCALAB 250Xi, Shimadzu Corporation, Kyoto, Japan). The microstructure of the VHCF powder was observed by means of scanning electron microscopy (SEM) using a field-emission microscope (SUPRA 55 SAPPHIRE, Carl Zeiss Corporation, Jena, Germany). The element distribution in the VHCF sample was detected by the above field-emission microscope equipped with an energy dispersive spectrometer (EDS). The microstructure of the sample was checked by means of transmission electron microscopy (TEM) and high-resolution TEM (HRTEM) measurements using an electron microscope (JEOL: 2100F, Joel Corporation, Tokyo, Japan). The thermal gravimetric (TG) curve was conducted using a thermal gravimetric analyzer (Mettler-Toledo, Zurich, Switzerland) in the air at a heating speed of 10 °C min^−1^.

### 2.3. Electrochemical Experiments

The electrochemical performance was tested using the LAND CT2001 battery tester. The voltage window of the electrochemical tests was 0.2–2.0 V. Coin cells (2032) were used to test the electrochemical properties of VHCF. A piece of Zn metal foil (100 μm thick) polished with sandpaper (2000 mesh) was used as the anode. The cathode was composed of VHCF powder at 70 wt%, Super P at 20 wt%, and a polyvinylidene fluoride (PVDF) binder at 10 wt%. To prepare the cathode electrodes, a slurry of VHCF, Super P, and PVDF was ground in N methyl-2-pyrrolidone (NMP) and then pasted onto a stainless-steel current collector. The electrode was dried in an oven at 60 °C for one day and cut into a circle using a mechanical punching machine. The diameter of the electrode is 8 mm. The loading mass of VHCF on the cathode was ~1 mg cm^−2^. A glass fiber filter (Whatman GF/C) was used as the separator. The electrolytes were 3 M Zn(CF_3_SO_3_)_2_ and 3 M Zn(CF_3_SO_3_)_2_ + 10 M LiTFSI aqueous solutions.

## 3. Results and Discussion

**Figure 1:**
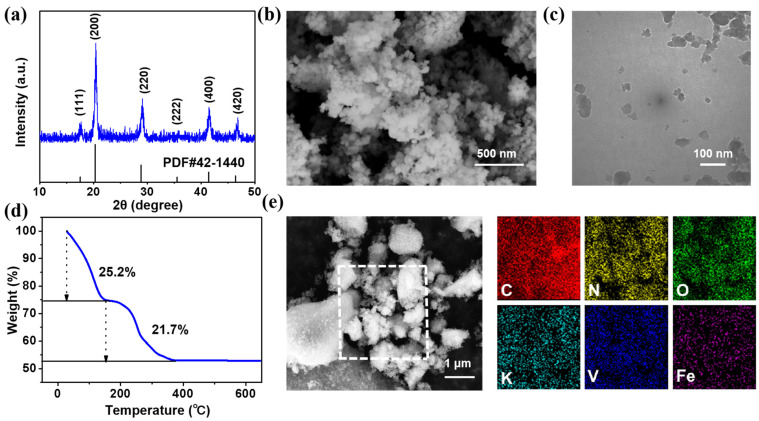
(**a**) XRD pattern, (**b**) SEM image, (**c**) TEM image, (**d**) TG curves, and (**e**) EDS images of VHCF.

As shown in Figure 1a, all of the identified XRD peaks of the sample were indexed to the cubic phase of V_1_._5_Fe(CN)_6_ (JCPDS No. 42-1440). Although the accurate crystal structure of VHCF is not well-studied in the literature so far, it can be expected that VHCF may exhibit similar properties to PBAs. In the VHCF crystallographic structure, V and Fe are connected by a shared CN ligand [26]. The formed porous 3D framework structure and large interstitial space can facilitate the reversible intercalation/de-intercalation of foreign cations. As shown in Figure 1b, VHCF consists of many irregular nanoparticles. The TEM image (Figure 1c) further shows that these nanoparticles are between 20 and 50 nm in size. Figure 1d is the TG curve of VHCF. The weight loss of the sample can be divided into two stages: the first stage below 200 °C is due to the evaporation of interstitial water (25.2%), and the second stage from 200 °C to 400 °C is ascribed to the composite decomposition of VHCF (21.7%) [26,27]. The EDS images (Figure 1e) confirm the even distribution of C, N, O, K, V, and Fe elements in VHCF. According to the inductively coupled plasma optical emission spectroscopy (ICP-OES) result, the molar ratio of K/V/Fe is 4.6/1.3/1.0, indicating that the possible chemical formula of VHCF is K_14_V_4_O_9_[Fe(CN)_6_]_3_·21.4H_2_O. In the VHCF crystallographic structure, 1/6 of the C=N groups are replaced by O atoms due to the presence of V=O ions, and part of the Fe(CN)_6_ complex sites are vacant because of the non-stoichiometry of V and Fe ions. This results in the formation of a large number of vacancies in VHCF, which are occupied by oxygen-terminating bonding. The massive void spaces and vacancies in the VHCF structure can accommodate large amounts of K ions and interstitial water molecules.

**Figure 2:**
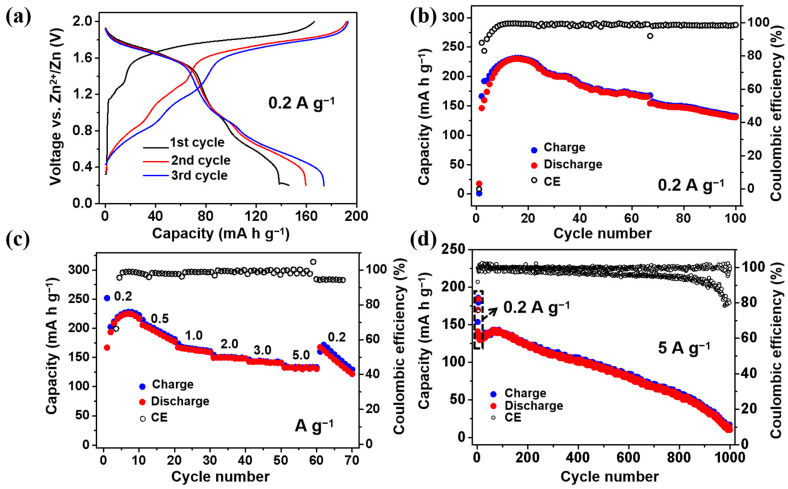
Electrochemical performance of VHCF in the 3 M Zn(CF_3_SO_3_)_2_ electrolyte; (**a**) The initial three charge/discharge profiles; (**b**) Cycling performance; (**c**) Rate performance; (**d**) Long-cycling stability.

The electrochemical properties of VHCF were first studied using 3 M Zn(CF_3_SO_3_)_2_ as the electrolyte. Figure 2a presents the initial three charge/discharge profiles of the VHCF cathode at 0.2 A g^−1^. The first discharge capacity is 146.2 mA h g^−1^, and it gradually increases to 174.0 mA h g^−1^ in the third cycle due to the activation process [28,29]. The operational voltage is about 1.2 V, which is much higher than that of other V-based cathode materials. The coulombic efficiencies of the first three cycles are 87.9%, 83.1%, and 90.1%, respectively. The low coulombic efficiency indicates that the battery may experience a loss of active material during cycling. As depicted in Figure 2b, the cycling performance of VHCF was evaluated at 0.2 A g^−1^. The discharge capacity gradually increases from 146.2 mA h g^−1^ to 230.3 mA h g^−1^ in 15 cycles, which is the highest capacity compared with other Zn//PBA batteries reported in the literature, indicating that the introduction of vanadium into the framework can greatly improve the redox activity of PBAs. However, the capacity retention after 100 cycles is only 56.7% (compared to the highest capacity reached during cycling). The fast capacity decay is most likely caused by the loss of VHCF during cycling. A rate capability test was performed to exhibit the capacities of the Zn//VHCF battery at higher current densities. As shown in Figure 2c, 221.5, 193.3, 163.3, 149.7, 142.9, and 133.6 mA h g^−1^ are obtained at 0.2, 0.5, 1.0, 2.0, 3.0, and 5.0 A g^−1^, respectively. It can be found that a high remaining capacity of 60.3% is achieved even with a 25-fold current density increase. Such a remarkable rate performance is mainly owing to the unique framework structure and intrinsic fast reaction kinetics of VHCF. Then, the long-cycling stability of VHCF was investigated at 5 A g^−1^ (Figure 2d, the first three cycles were performed at 0.2 A g^−1^). The cell shows a negligible capacity of 10.1 mA h g^−1^ after 1000 cycles, resulting in a low capacity retention of 7.1%. In addition, its coulombic efficiency gradually decreases to 80% after 1000 cycles. These results suggest that VHCF is a promising cathode material for AZIBs due to its excellent Zn^2+^ storage capability. However, the bad cyclic stability of VHCF in the conventional 3 M Zn(CF_3_SO_3_)_2_ electrolyte limits its practical application. The cycling performance of the Zn//VHCF battery was also tested with a narrow voltage window between 1.2 V and 2.0 V. As shown in Figure S1, the battery shows good cycling stability. This indicates that the limited cycling life of the Zn//VHCF battery with a low cutoff voltage of 0.2 V is probably due to the dissolution of the active material.

**Figure 3:**
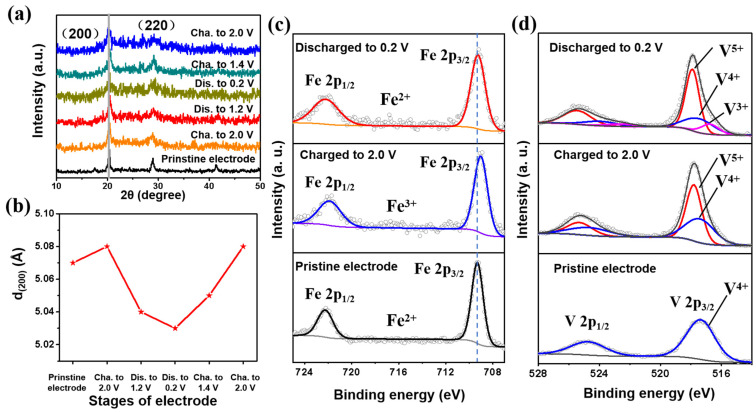
(**a**) XRD curves and (**b**) (200) interplanar spacing variations of VHCF at different charge/discharge stages of the first cycle; (**c**) Fe 2p and (**d**) V 2p XPS regions for the pristine, charged, and discharged VHCF electrodes.

The Zn^2+^ storage mechanism of VHCF was investigated in depth by XRD characterization and XPS analysis. Figure 3a shows the XRD curves of VHCF at different charging/discharging stages and Figure 3b depicts the (200) interplanar spacing variations calculated by the Bragg formula. It can be observed that the (200) diffraction peak shifts slightly to a lower angle with interplanar spacing increasing from 5.07 Å to 5.08 Å after first being charged to 2.0 V along with the de-intercalation of K^+^ ions. During the subsequent discharge and charge, the interplanar spacing shrinks from 5.08 Å to 5.03 Å and then returns to the value after the first charge, indicating the reversible structure change of the VHCF electrode. It can be found that the (200) interplanar spacing expansions and contractions are very small (~1%), with almost zero strain [30]. XPS was employed to further investigate the valence change of VHCF after the electrochemical reactions. As shown in Figure S2, the intensity of the K XPS peak decreases after being charged to 2.0 V, indicating that part of the K^+^ ions (not all of them) are extracted from the VHCF framework during the initial charging process. When the electrode is discharged to 0.2 V, the intensity of the K XPS peak is almost unchanged. It reveals that K^+^ will not participate in the following electrochemical reactions. Figure 3c shows the Fe 2p XPS region. It can be found that Fe^2+^ in VHCF is oxidized to Fe^3+^ during the first charge and reduced to Fe^2+^ after the first discharge. In addition, V can be another redox-active site of VHCF. As shown in Figure 3d, the initial valence of V in the VHCF electrode is 4+. When charged to 2.0 V, V^4+^ is partially oxidized to V^5+^, and the average valence of V becomes 4.72+. A new XPS peak appears at 516.7 eV after discharging to 0.2 V, which can be assigned to V^3+^, indicating that V is reduced with the intercalation of zinc ions. As shown in Figure S3, three charge/discharge plateaus can be observed in a typical charge/discharge profile. Plateau 1 between 1.2 V and 2.0 V is attributed to the redox of Fe ions. Plateau 2 from 0.6 V to 1.2 V and plateau 3 from 0.2 V to 0.6 V are attributed to the redox of V ions. The corresponding capacities from the Fe redox center (70 mA h g^−1^) and V redox center (160 mA h g^−1^) are consistent with the ratio of Fe and V in the chemical formula of VHCF. These results demonstrate that there are two electrochemical redox-active centers in the VHCF electrode (Fe^2+^ ⇋ Fe^3+^ and V^5+^ ⇋ V^4+^ ⇋ V^3+^) [31,32]. This enables the Zn//VHCF battery to achieve a three-electron-involved redox reaction, providing an ultra-high reversible capacity.

**Figure 4:**
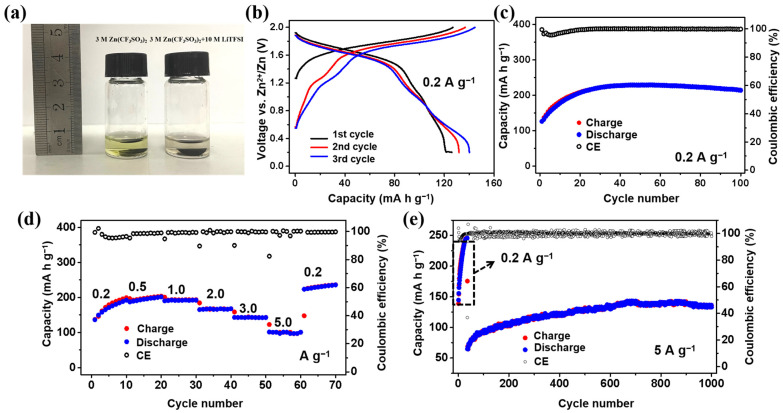
(**a**) Digital photo of the VHCF electrodes after immersion in two different electrolytes for 2 weeks at room temperature; (**b**) The initial three charge/discharge profiles; (**c**) cycling performance; (**d**) rate performance; and (**e**) long-cycling stability of VHCF in the 3 M Zn(CF_3_SO_3_)_2_ + 10 M LiTFSI electrolyte.

Although the VHCF cathode exhibits the advantages of high capacity and high voltage, its very bad cycling performance means that it is not competitive with other materials. The dissolution of active species in a dilute aqueous electrolyte is a common problem for PBA cathodes. To enhance the cycling performance of VHCF, a high-concentration hybrid electrolyte (3 M Zn(CF_3_SO_3_)_2_ + 10 M LiTFSI) was used. Figure 4a compares the dissolution of a VHCF electrode after immersion in two different electrolytes at room temperature for 2 weeks. It can be found that the 3 M Zn(CF_3_SO_3_)_2_ electrolyte turns yellow, while the 3 M Zn(CF_3_SO_3_)_2_ + 10 M LiTFSI electrolyte is still almost colorless and transparent. This demonstrates that the high-concentration electrolyte can prevent the dissolution of VHCF. As plotted in Figure 4b, the discharge capacities and the coulombic efficiencies of the first three cycles are 126.1 mA h g^−1^, 131.9 mA h g^−1^, 140.2 mA h g^−1^, and 99.3%, 96.3%, 97.0%, respectively. As shown in Figure 4c, the specific capacity of the VHCF electrode reaches the highest value of 228.8 mA h g^−1^ in 45 cycles. A capacity of 214 mA h g^−1^ is maintained after 100 cycles (93.5%). Although the first discharge capacity of VHCF in this electrolyte is lower, it can still reach almost the same capacity as that in 3 M Zn(CF_3_SO_3_)_2_ after an activation process. Figure S4 shows the charge/discharge profiles of VHCF in the 3 M Zn(CF_3_SO_3_)_2_ + 10 M LiTFSI electrolyte at 0.2 A g^−1^ after 20, 40, 60, 80, and 100 cycles, respectively. It can be found that the increase in capacity before the 45th cycle is mainly due to the elongation of the plateaus between 0.2 V and 1.2 V. It indicates that the redox reactions of V ions in VHCF are gradually activated during the initial cycling stage. However, the rate capability of the cell is slightly reduced, mainly caused by the lower ion conductivity of the high-concentration electrolyte (Figure 4d). The recorded capacities are 171.0, 194.7, 191.5, 166.1, 143.2, and 100.9 mA h g^−1^ at 0.2, 0.5, 1.0, 2.0, 3.0, and 5.0 A g^−1^, respectively. Nevertheless, its energy/power densities at 5.0 A g^−1^ (81.0 Wh Kg^−1^/4013.9 W Kg^−1^) are still higher than that of the traditional PBA cathodes in AZIBs [33,34,35]. Moreover, it shows a high capacity retention of 92.9% with an average efficiency of ~100% after 1000 cycles (Figure 4e). As shown in Figure S5, the surfaces of the VHCF electrodes are covered with a film after 1000 cycles, which may be a solid electrolyte interface (SEI) film. It can be found that the film on the VHCF electrode surface in 3 M Zn(CF_3_SO_3_)_2_ +10 M LiTFSI after 1000 cycles is denser and more uniform, which can more efficiently inhibit the dissolution of active material. The above results suggest that the Zn//VHCF batteries using the high-concentration hybrid electrolyte have the advantages of high capacity, high voltage, and excellent long-cycling stability, and are expected to become the next generation of safe and cheap large-scale energy storage batteries.

## 4. Conclusions

In summary, VHCF powders can be easily prepared and are suitable for AZIBs as the cathode material. The VHCF cathode exhibits an impressive capacity of 230.3 mA h g^−1^ and a high average voltage of ~1.2 V. Experimental investigations demonstrate that the high capacity comes from the two electrochemical redox-active centers in VHCF (Fe^2+^ ⇋ Fe^3+^ and V^5+^ ⇋ V^4+^ ⇋ V^3+^), and the intercalation/de-intercalation of Zn^2+^ can occur without VHCF phase transition at almost zero lattice strain. The fast capacity decay in the 3 M Zn(CF_3_SO_3_)_2_ electrolyte is due to the loss of VHCF. The cycling performance of the Zn//VHCF cell can be largely improved by using a high-concentration hybrid electrolyte that can inhibit the dissolution of VHCF. The improved battery exhibits a high capacity of 235.8 mA h g^−1^ and a remarkable capacity retention of 92.9% after 1000 cycles. These findings not only contribute to understanding the electrochemical behaviors of PBA cathodes in AZIBs, but also have important implications for the development of more high-performance cathode materials for cost-efficient and safe energy storage systems. However, the detailed electrochemical mechanisms of VHCF, especially the structure evolution and the role of K^+^ ions during the charge/discharge process require further studies. These issues will become a focus of our future research.

## Data Availability

The data that support the findings of this study are available from the corresponding authors upon reasonable request.

## Supplementary Materials

The following are available online at https://www.mdpi.com/article/10.3390/nano12234268/s1, Figure S1: The cycling performance of the Zn//VHCF battery in 3 M Zn(CF_3_SO_3_)_2_ electrolyte at 0.2 A g^−1^ with a voltage window between 1.2 V and 2.0 V; Figure S2: K 1s XPS regions for the pristine, charged and discharged VHCF electrodes; Figure S3: The charge/discharge profiles of the VHCF//Zn battery in 3 M Zn(CF_3_SO_3_)_2_ electrolyte after 15 cycles at 0.2 A g^−1^; Figure S4: The charge/discharge profiles of VHCF in 3 M Zn(CF_3_SO_3_)_2_ + 10 M LiTFSI electrolyte at 0.2 A g^−1^ after 20, 40, 60, 80 and 100 cycles, respectively; Figure S5: The FESEM images of (a) the pristine VHCF electrodes, (b) the VHCF electrode in 3 M Zn(CF_3_SO_3_)_2_ after 1000 cycles, and (c) the VHCF electrode in 3 M Zn(CF_3_SO_3_)_2_ +10 M LiTFSI after 1000 cycles.
