# Supplementary material for: Vanadium Hexacyanoferrate as a High-Capacity and High-Voltage Cathode for Aqueous Rechargeable Zinc Ion Batteries
Source: Nanomaterials (Basel). 2022 Nov 30;12(23):4268. doi: 10.3390/nano12234268 (PMC9740437; doi:10.3390/nano12234268)
Supplement: Supplementary file 1 [file nanomaterials-12-04268-s001.zip › nanomaterials-2042831-supplementary.pdf]

Supplementary Materials

Vanadium Hexacyanoferrate as a High-Capacity and High-Voltage Cathode for Aqueous Rechargeable Zinc Ion Batteries

Shijing Zhang [1], Qiang Pang [1,*], Yuqing Ai [1], Wei He [1], Yao Fu [1], Mingming Xing [1], Ying Tian [1,*] and Xixian Luo [2]

[1] School of Science, Dalian Maritime University, Dalian 116026, China
[2] School of Physics and Materials Engineering, Dalian Minzu University, Dalian 116600, China
* Correspondence: pangqiang@dlmu.edu.cn (Q.P.); tianying@dlmu.edu.cn (Y.T.)

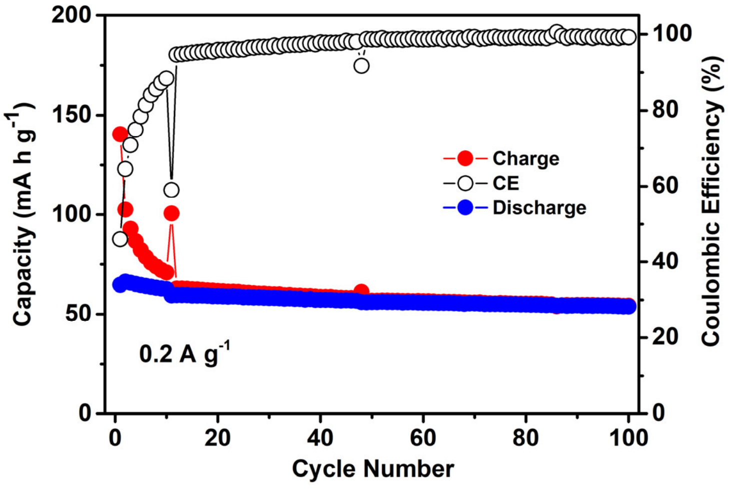

Figure S1. The cycling performance of the Zn//VHCF battery in 3 M $Zn(CF_3SO_3)_2$ electrolyte at 0.2 A g^{-1} with a voltage window between 1.2 V and 2.0 V.

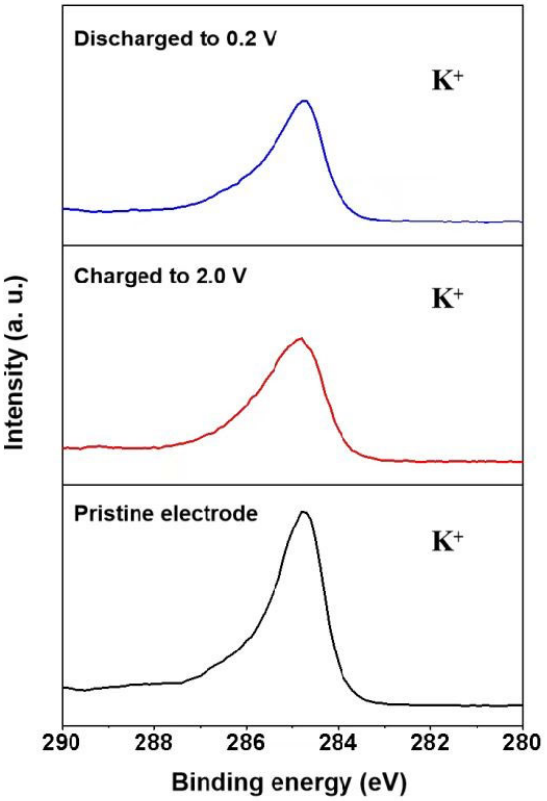

Figure S2. K 1s XPS regions for the pristine, charged and discharged VHCF electrodes.

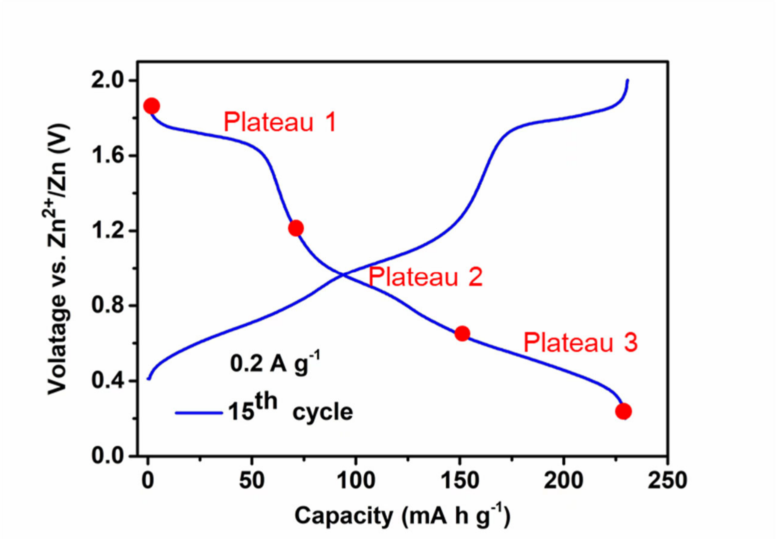

Figure S3. The charge/discharge profiles of the VHCF//Zn battery in 3 M $Zn(CF_3SO_3)_2$ electrolyte after 15 cycles at 0.2 A g^{-1}.

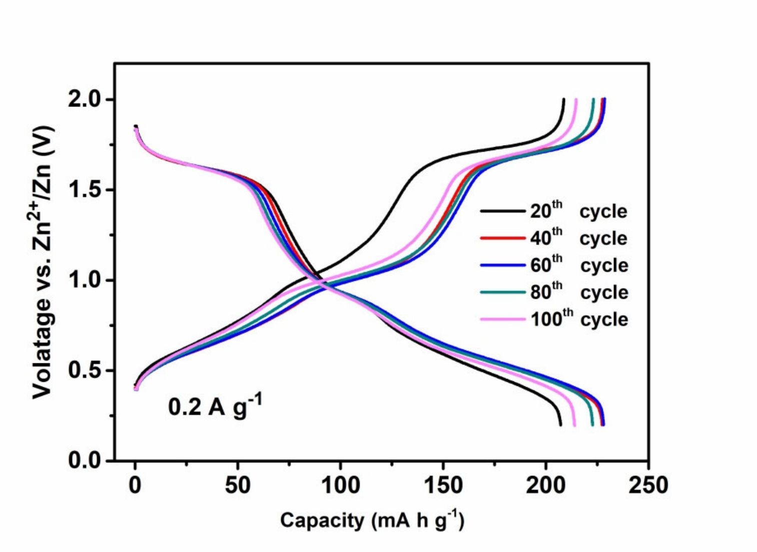

Figure S4. The charge/discharge profiles of VHCF in 3 M $Zn(CF_3SO_3)_2$ + 10 M LiTFSI electrolyte at 0.2 A g^{-1} after 20, 40, 60, 80 and 100 cycles, respectively.

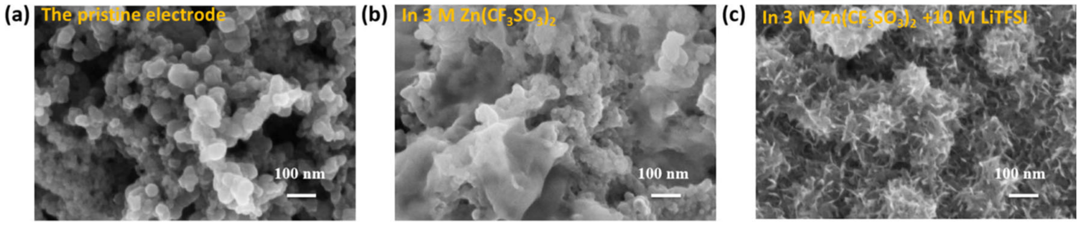

Figure S5. The FESEM images of (**a**) the pristine VHCF electrodes, (**b**) the VHCF electrode in 3 M Zn(CF3SO3)2 after 1000 cycles, and (**c**) the VHCF electrode in 3 M Zn(CF3SO3)2 +10 M LiTFSI after 1000 cycles.
